# PTEN participates in airway remodeling of asthma by regulating CD38/Ca^2+^/CREB signaling

**DOI:** 10.18632/aging.103664

**Published:** 2020-08-27

**Authors:** Yuanyuan Wu, Yiyi Lu, Fan Zou, Xinping Fan, Xudong Li, Hongni Zhang, Haijuan Chen, Xiuzhen Sun, Yun Liu

**Affiliations:** 1 Department of Respiratory and Critical Care Medicine, The Second Affiliated Hospital of Xi’an Jiaotong University, Xi’an 710004, Shaanxi Province, PR China

**Keywords:** airway remodeling, asthma, CREB, CD38, PTEN

## Abstract

Both phosphatase and tensin homologue deleted on chromosome ten (PTEN) and cluster of differentiation 38 (CD38) have been suggested to be key regulators of the pathogenesis of asthma. However, the precise role and molecular mechanisms by which PTEN and CD38 are involved in airway remodeling throughout asthma pathogenesis remains poorly understood. This study aimed to elucidate the role of PTEN and CD38 in airway remodeling of asthma. Exposure to tumor necrosis factor-α (TNF-α) in airway smooth muscle (ASM) cells markedly decreased PTEN expression, and increased expression of CD38. Overexpression of PTEN suppressed the expression of CD38 and downregulated proliferation and migration induced by TNF-α stimulation, which was partially reversed by CD38 overexpression. PTEN/CD38 axis regulated Ca2+ levels and cyclic AMP response-element binding protein (CREB) phosphorylation in TNF-α-stimulated ASM cells. The in vitro knockdown of CD38 or overexpression of PTEN remarkably restricted airway remodeling and decreased Ca2+ concentrations and CREB phosphorylation in asthmatic mice. CD38 overexpression abolished the inhibitory effects of PTEN overexpression on airway remodeling. These findings demonstrate that PTEN inhibits airway remodeling of asthma through the downregulation of CD38-mediated Ca2+/CREB signaling, highlighting a key role of PTEN/CD38/Ca2+/CREB signaling in the molecular pathogenesis of asthma.

## INTRODUCTION

Asthma is one of most common chronic diseases that negatively affects the quality of life of patients and greatly impacts health care costs globally [1, 2]. Understanding airway remodeling, a hallmark of asthma, has been suggested to be important for understanding the pathogenesis of asthma [3, 4]. Airway smooth muscle (ASM) cells are the central players involved in airway remodeling in patients with asthma [5]. The hyperproliferation and excessive migration of ASM cells are stimulated by inflammatory factors that contribute to hyperplasia and increased ASM mass, and result in airway remodeling in asthma [5]. Therefore, understanding the molecular mechanism of airway remodeling could facilitate the development of novel and promising therapeutic strategies for the treatment of asthma.

Phosphatase and tensin homologue deleted on chromosome ten (PTEN) is a lipid phosphatase that plays a key role in multiple signal transduction pathways [6]. PTEN is involved in regulating cellular processes including cell survival, proliferation and migration, and participates in diverse physiological and pathological processes [6]. Notably, PTEN has emerged as a crucial regulator of the pathogenesis of asthma [7]. PTEN expression is decreased in lung tissues of asthmatic mice and intratracheal overexpression of PTEN markedly restricts the bronchial inflammation and airway hyperresponsiveness of asthmatic mice [8]. Moreover, PTEN is involved in regulating the proliferation and migration of ASM cells, and thereby contributes to airway remodeling in asthma [9–13]. However, the precise molecular mechanism by which PTEN regulates airway remodeling in asthma remains unclear.

Cluster of differentiation 38 (CD38) is a multifunctional protein that plays a key role in mobilizing intracellular Ca^2+^ [14]. CD38 is an important mammalian adenosine diphosphate (ADP)-ribosyl cyclase that catalyzes the production of cADPR using nicotinamide adenine dinucleotide, which regulates the mobilization and entry of Ca^2+^ [15, 16]. CD38/Ca^2+^ signaling affects a wide range of biological processes, which include proliferation and differentiation [17]. Interestingly, CD38 has been suggested as a key regulator of asthma [18]. CD38 is ubiquitously expressed in ASM and its expression is augmented in asthma [19]. In CD38 deficient mice, airway hyperresponsiveness induced by interleukin (IL)-13 or tumor necrosis factor (TNF)-α is reduced [20, 21]. Therefore, CD38 has emerged as an attractive drug target for the treatment of asthma.

Cyclic AMP response-element binding protein (CREB) is a critical transcriptional factor that promotes the expression of various target genes via binding to a conserved cAMP response-element in its promoter region [22]. CREB activation regulates various cellular processes such as proliferation and migration [23, 24]. Notably, recent studies have reported that CREB activation enhances proliferation of ASM cells [24–26]. Therefore, CREB may play an important role in airway remodeling in asthma.

Although PTEN and CD38 have been reported to have crucial roles in the pathogenesis of asthma, the precise molecular mechanisms of PTEN and CD38 in airway remodeling throughout the pathogenesis of asthma remain poorly understood. In the present study, we explore the role of PTEN and CD38 in airway remodeling in asthma both *in vitro* and *in vivo*. Our findings demonstrate that PTEN inhibits ASM proliferation and migration *in vitro* and airway remodeling *in vivo* through the downregulation of CD38-mediated Ca^2+^/CREB signaling, which underscores the a key role of PTEN/CD38/Ca^2+^/CREB signaling in the molecular pathogenesis of asthma.

## RESULTS

### Effect of TNF-α stimulation on PTEN and CD38 expression in ASM cells

**Figure 1:**
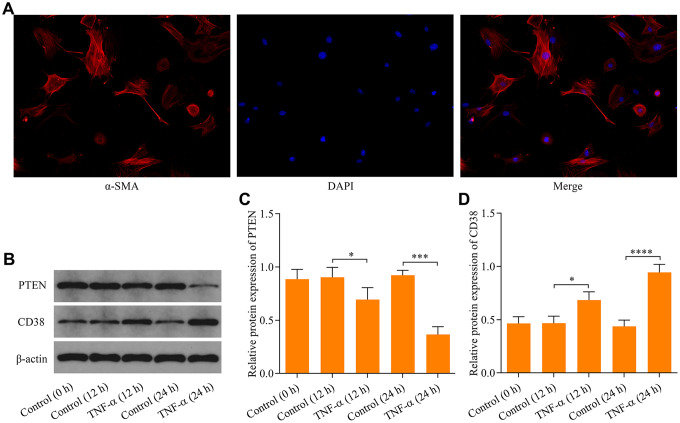
**Effect of TNF-α stimulation on PTEN and CD38 expression in ASM cells.** (**A**) Identification of ASM cells by immunocytochemical staining with α-SMA (magnification: 200X). ASM cells were stimulated with 20 ng × ml^-1^ TNF-α and incubated for either 12 h or 24 h, and (**B**) protein expression of PTEN and CD38 were examined using Western blot. Quantitative analysis of (**C**) PTEN and (**D**) CD38 protein expression shown in B (n = 3, *p < 0.05, ***p < 0.001 and ****p < 0.0001).

The effect of TNF-α stimulation on expression of PTEN and CD38 in ASM cells were investigated initially using primary mouse ASM cells (Figure 1A). Exposure of ASM cells to TNF-α caused a significant decrease in PTEN expression (Figure 1B, 1C), while a marked increase in CD38 expression was observed (Figure 1B, 1D). These data indicate that TNF-α stimulation alters PTEN and CD38 expression in ASM cells.

### Knockdown of CD38 impedes TNF-α-induced proliferation and migration of ASM cells

**Figure 2:**
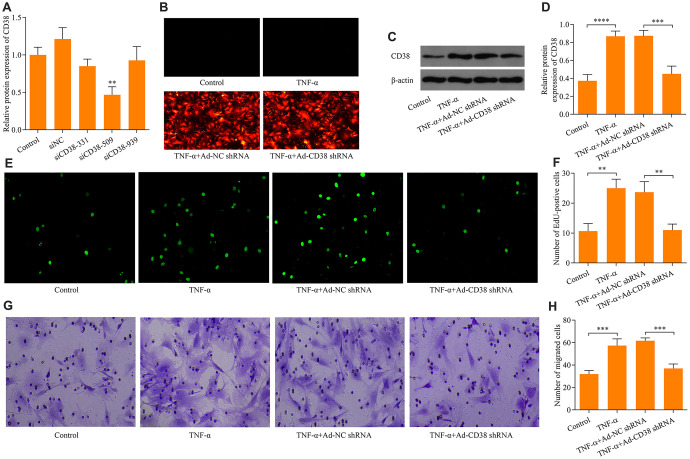
**Knockdown of CD38 impedes TNF-α-induced proliferation and migration in ASM cells**. (**A**) RT-qPCR detection was used to determine knockdown efficacy of three designed CD38 siRNAs (siCD38-331, siCD38-509 and siCD38-939; n = 3, **p < 0.01). (**B**) Determination of recombinant adenovirus infection efficacy via GFP fluorescence using immunocytochemistry (magnification: 200X). ASM cells were infected with Ad-CD38 shRNA or Ad-NC shRNA for 48 h and then stimulated with 20 ng × ml^-1^ TNF-α for 24 h, and (**C**, **D**) protein expression of CD38 was determined via Western blot (n = 3, ***p < 0.001 and ****p < 0.0001). (**E**, **F**) The effect of CD38 knockdown on TNF-α-induced ASM cell proliferation evaluated via EdU proliferation assay (magnification: 200X) (n = 3, **p < 0.01). (**G**, **H**) The effect of CD38 knockdown on TNF-α-induced ASM cell migration assessed by Transwell migration assay (magnification: 200X) (n = 3, ***p < 0.001).

To investigate the precise effect of CD38 on the regulation of TNF-α-induced proliferation and migration of ASM cells, we performed experiments examining the effects of the loss-of-function of CD38. Three siRNAs targeting CD38 were designed and their knockdown efficacy was determined using RT-qPCR. The results demonstrated that siCD38-509 more efficiently knocked down expression of CD38 than the other two CD38-targeted siRNAs (Figure 2A). Therefore, siCD38-509 was used to design CD38 shRNA sequences. The designed CD38 shRNA was subcloned into adenovirus vectors to produce recombinant adenovirus expressing CD38 shRNA (Ad-CD38 shRNA). Recombinant adenovirus was used to infect ASM cells and infection efficiency was determined by observing the RFP fluorescence of the adenovirus vector (Figure 2B). Western blot analysis confirmed that infection of Ad-CD38 shRNA markedly decreased the protein expression of CD38 in ASM cells (Figure 2C, 2D). Notably, knockdown of CD38 inhibited TNF-α-induced proliferation of ASM cells (Figure 2E, 2F). Moreover, knockdown of CD38 significantly blocked TNF-α-induced ASM cell migration (Figure 2G, 2H). Collectively, these data suggest that CD38 knockdown restricts TNF-α-induced proliferation and migration of ASM cells *in vitro*.

### PTEN suppresses TNF-α-induced ASM cell proliferation and migration by downregulating CD38 expression

**Figure 3:**
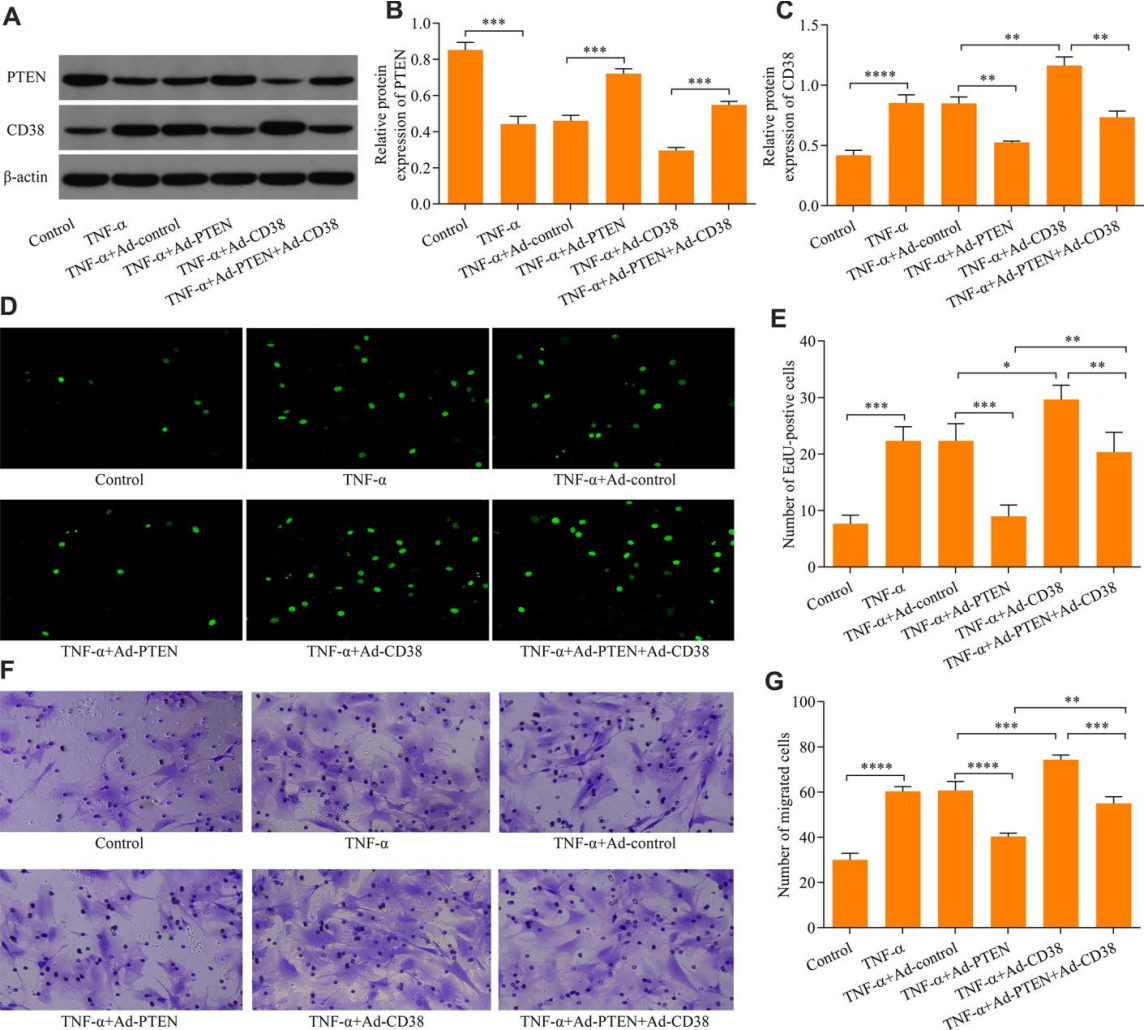
**PTEN suppresses TNF-α-induced ASM cell proliferation and migration by CD38.** ASM cells were infected with Ad-PTEN and/or Ad-CD38 and incubated for 48 h, which was followed by stimulation with o20 ng × ml^-1^ TNF-α for 24 h, and (**A**) protein expression of PTEN and CD38 was examined via Western blot. Quantitative analysis of the expression of the (**B**) PTEN and (**C**) CD38 protein determined in A (n = 3, **p < 0.01, ***p < 0.001, ****p < 0.0001). (**D**, **E**) Cell proliferation assessed using an EdU proliferation assay (magnification: 200X) (n = 3, *p < 0.05, **p < 0.01, ***p < 0.001,). (**F**, **G**) Cell migration evaluated using a Transwell migration assay (magnification: 200X) (n = 3, **p < 0.01, ***p < 0.001, ****p < 0.0001).

To determine the regulatory role of PTEN on CD38 expression, we assessed the effects of PTEN gain-of-function. ASM infection with Ad-PTEN resulted in significant increases of PTEN protein expression (Figure 3A, 3B). Moreover, PTEN overexpression markedly downregulated the CD38 expression induced by TNF-α stimulation in ASM cells (Figure 3A–3C). Notably, PTEN overexpression markedly impeded TNF-α-induced ASM cell proliferation (Figure 3D, 3E) and migration (Figure 3F, 3G). By contrast, CD38 overexpression exacerbated TNF-α-induced ASM cell proliferation and migration, and reversed the suppressive effect of PTEN overexpression on TNF-α-induced ASM cell proliferation and migration (Figure 3D–3G). Overall, these findings indicate that PTEN overexpression suppresses TNF-α-induced ASM cell proliferation and migration via the downregulation of CD38.

### PTEN blocks TNF-α-induced Ca2+ accumulation and CREB activation by downregulating CD38

**Figure 4:**
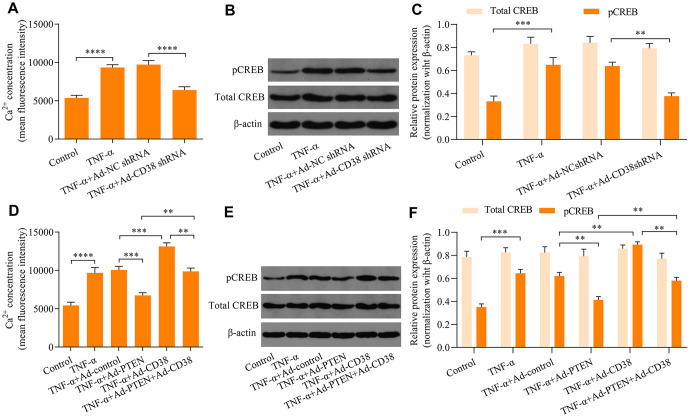
**PTEN blocks TNF-α-induced Ca^2+^ accumulation and CREB activation by downregulating CD38**. ASM cells infected with Ad-CD38 shRNA were incubated for 48 h and stimulated with of 20 ng × ml^-1^ TNF-α for 24 h, and (**A**) intracellular Ca^2+^ concentrations were measured with Fluo-3 AM fluorescent probes, and (**B**, **C**) the protein expression patterns of total CREB and phosphorylated CREB (pCREB) were determined via Western blot (n = 3, **p < 0.01 ***p < 0.001 and ****p < 0.0001). ASM cells were infected with Ad-PTEN or/and Ad-CD38, incubated for 48 h, and stimulated using of 20 ng × ml^-1^ TNF-α for 24 h. (**D**) Intracellular Ca^2+^ concentrations were assessed using Fluo-3 AM fluorescent probes, and (**E**, **F**) protein expression levels of total CREB and phosphorylated CREB (pCREB) were determined via Western blot (n = 3, **p < 0.01 ***p < 0.001 and ****p < 0.0001).

To further explore the molecular mechanisms of the PTEN/CD38 axis in the regulation of TNF-α-induced ASM cell proliferation and migration, we assessed regulatory effects of PTEN/CD38 on Ca2+ levels induced by TNF-α stimulation. We found that knockdown of CD38 significantly downregulated the Ca2+ accumulation induced by TNF-α stimulation (Figure 4A). Notably, TNF-α stimulation-induced phosphorylation of CREB also decreased as a result of CD38 knockdown (Figure 4B, 3C). Moreover, PTEN overexpression markedly attenuated the induction of Ca2+ levels and CREB phosphorylation by TNF-α stimulation (Figure 4D–4F). In contrast, CD38 overexpression exacerbated Ca2+ increases, enhanced CREB phosphorylation induced by TNF-α stimulation, and reversed PTEN overexpression-mediated suppressive effects mediating TNF-α-induced Ca2+ increases and CREB activation (Figure 4D–4F). Collectively, these data suggest that PTEN blocks TNF-α-induced increases in Ca2+ levels and CREB activation by downregulating CD38.

### PTEN and CD38 expression within the lung tissue of asthmatic mice

**Figure 5:**
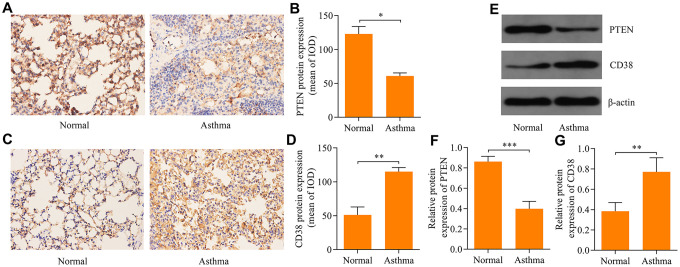
**Expression of PTEN and CD38 in lung tissues of asthmatic mice.** Immunohistochemistry used to assess expression of (**A**, **B**) PTEN and (**C**, **D**) CD38 proteins in the lung tissue of asthmatic mice (magnification: 400X). (**E**) Western blot used to assess protein expression levels of PTEN and CD38 in the lung tissue of asthenic mice. Quantitative analysis of (**F**) PTEN and (**G**) CD38 protein expression determined in E (n = 3, *p < 0.05, **p < 0.01 and ***p < 0.001).

To confirm the involvement of PTEN and CD38 in the pathogenesis asthma, we examined protein expression levels of PTEN and CD38 in the lung tissue of asthmatic mice using immunohistochemistry. Results revealed that PTEN expression decreased (Figure 5A, 5B), while CD38 expression was elevated (Figure 5C, 5D) in the lung tissue of asthmatic mice. Moreover, Western blot confirmed that levels of PTEN and CD38 expression were reduced and elevated, respectively, in lung tissues of asthmatic mice (Figure 5E–5G). Therefore, these data confirm the involvement of PTEN and CD38 in asthma.

### CD38 knockdown inhibits airway remodeling in asthmatic mice *in vivo*

**Figure 6:**
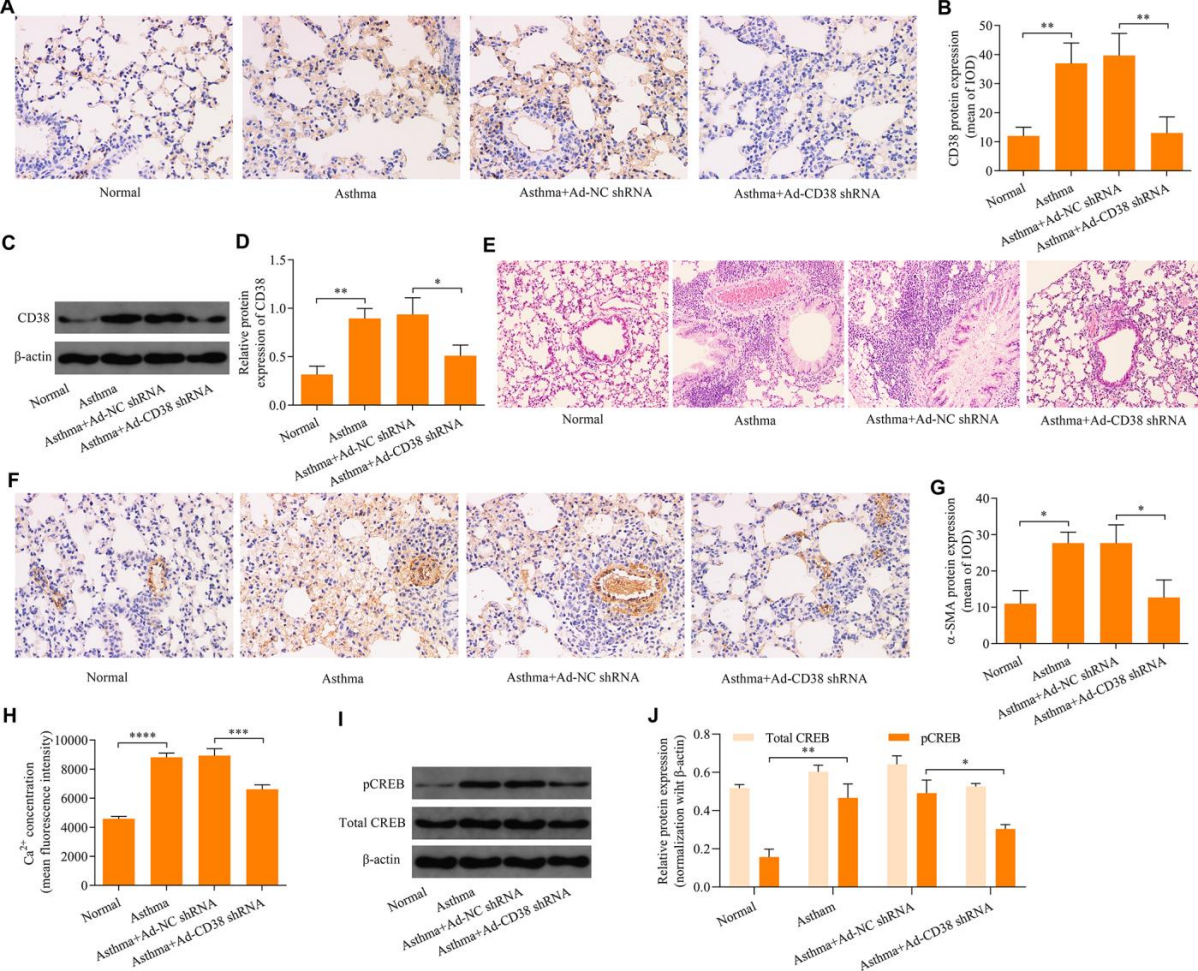
**Knockdown of CD38 inhibits airway remodeling in asthmatic mice.** (**A**, **B**) Immunohistochemistry analysis of CD38 protein expression in lung tissues. (**C**, **D**) Western blot analysis of CD38 protein expression in lung tissues (magnification: 400X). (**E**) Hemolysin staining of lung tissues (magnification: 400X). (**F**, **G**) Determination of ASM cell mass via immunohistochemistry staining of α-SMA (magnification: 400X). (**H**) Intracellular Ca^2+^ concentrations measured via Fluo-3 AM fluorescent probes in lung tissues. (**I**, **J**) Protein expression of total CREB an pCREB determined using Western blot. n = 3, *p < 0.05, **p < 0.01, ***p < 0.001 and ****p < 0.0001.

To explore the effect of CD38 in the regulation of airway remodeling in asthma, we downregulated CD38 expression in the lung tissue of asthmatic mice via intratracheal injection of adenovirus-expressing CD38 shRNA. Downregulation of CD38 in the lung tissue of mice was confirmed using immunohistochemistry (Figure 6A, 6B) and Western blot (Figure 6C, 6D) analyses. HE staining of lung tissue revealed that mice infected with Ad-CD38 shRNA exhibited reductions in airway epithelium thickening and inflammatory cell infiltration within peribronchiolar tissue (Figure 6E). Notably, immunohistochemistry analysis revealed that that CD38 knockdown was associated with decreased ASM cell mass in the lung tissues of asthmatic mice (Figure 6F, 6G). Moreover, knockdown of CD38 significantly decreased the induction of Ca^2+^ (Figure 6H) and CREB phosphorylation (Figure 6I–6J) in lung tissues of asthmatic mice. Taken together, these results indicate that CD38 knockdown inhibits airway remodeling in asthmatic mice and is associated with downregulation of the induction of Ca^2+^ and CREB phosphorylation.

### PTEN suppresses airway remodeling in asthma through regulation of CD38

**Figure 7:**
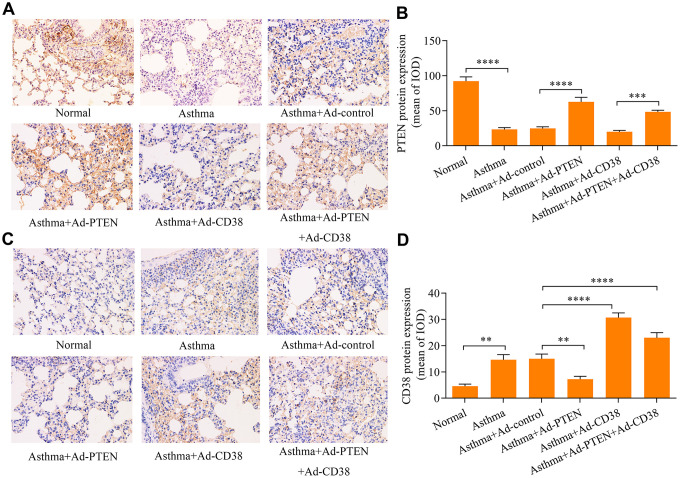
**Immunohistochemistry used to assess PTEN and CD38 expression in the lung tissues of asthmatic mice infected with Ad-PTEN and/or Ad-CD38.** (**A**, **B**) Expression of PTEN and (**C**, **D**) CD38 proteins in lung tissues were examined via immunohistochemistry analyses (magnification: 400X) (n = 3, **p < 0.01, ***p < 0.001, ****p<0.0001).

**Figure 8:**
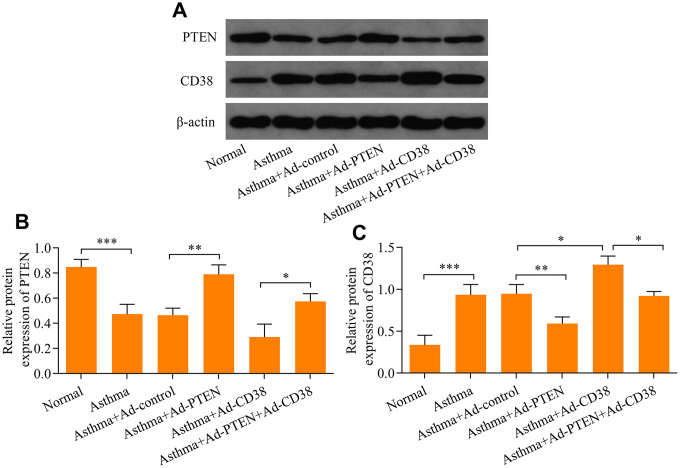
**Western blot analysis of PTEN and CD38 expression in the lung tissue of asthmatic mice infected with Ad-PTEN or/and Ad-CD38**. (**A**) Protein expression of PTEN and CD38 determined using Western blot. Quantitative analysis of (**B**) PTEN and (**C**) CD38 protein expression determined in A (n = 3, *p < 0.05, **p < 0.01, and ***p<0.001).

**Figure 9:**
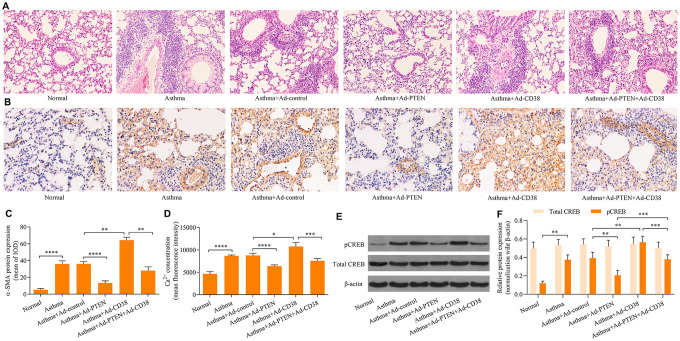
**CD38 overexpression reverses PTEN-mediated suppressive effects on airway remodeling, Ca^2+^ induction and CREB phosphorylation in the lung tissue of asthmatic mice.** (**A**) HE staining of lung tissues (magnification: 400X). (**B**, **C**) Detection of ASM cell mass using immunohistochemistry staining of α-SMA (magnification: 400X). (**D**) Intracellular Ca^2+^ concentrations measured via Fluo-3 AM fluorescent probes in lung tissues. (**E**, **F**) Expression of total CREB and pCREB protein levels were determined using Western blot. n = 3, *p < 0.05, **p < 0.01, ***p < 0.001 and ****p<0.0001.

We further determined the role of PTEN in airway remodeling throughout the pathogenesis of asthma. We found that Ad-PTEN infection significantly enhanced expression of the PTEN protein in the lung tissue of asthmatic mice, and caused a marked reduction in the expression of the CD38 protein (Figure 7A–7D and Figure 8A–8C). Moreover, PTEN overexpression markedly attenuated airway remodeling, while CD38 overexpression exacerbated airway remodeling in asthmatic mice (Figure 9A–9C). Notably, CD38 overexpression attenuated PTEN overexpression-mediated suppressive effects on airway remodeling in asthmatic mice (Figure 9A–9C). In addition, PTEN overexpression was associated with decreased Ca^2+^ induction and CREB phosphorylation in the lung tissue of asthmatic mice, while CD38 overexpression produced opposite effects (Figure 9D–9F). CD38 overexpression also significantly reduced PTEN overexpression-mediated inhibitory effects on Ca^2+^ induction and CREB phosphorylation in the lung tissue of asthmatic mice (Figure 9D–9F). Collectively, these data indicate that PTEN suppresses airway remodeling, which is associated with the attenuation of Ca^2+^ induction and CREB phosphorylation via inhibition of CD38.

## DISCUSSION

**Figure 10:**
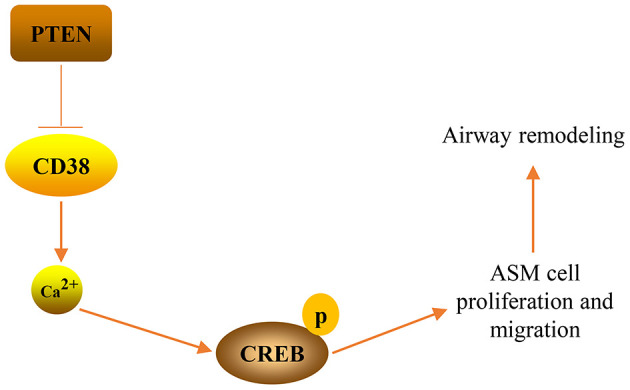
**A graphical model of the PTEN/CD38/Ca^2+^/CREB axis that regulates airway remodeling in asthma.**

In the present study, we have shown that the PTEN/CD38/Ca^2+^/CREB axis participates in airway remodeling in asthma. *In vitro* experiments revealed that PTEN impeded TNF-α-induced proliferation and migration of ASM cells by downregulating CD38-meidated Ca^2+^/CREB signaling. Notably, *in vivo* experiments confirmed that PTEN overexpression, or CD38 knockdown, decreased airway remodeling associated with the downregulation of Ca^2+^/CREB signaling in the lung tissue of asthmatic mice. Taken together, the results of our study have shown that the PTEN/CD38/Ca^2+^/CREB axis plays a critical role in airway remodeling that occurs throughout the progression of asthma (Figure 10).

PTEN has emerged as a key modulator of asthma pathogenesis [7]. PTEN expression is decreased in the lung tissues of asthmatic mice and the intratracheal overexpression of PTEN markedly restricts bronchial inflammation and airway hyperresponsiveness in asthmatic mice [8]. Moreover, PTEN is involved in regulating airway remodeling in asthma. Overexpression of PTEN decreases the proliferative and migrative capabilities of ASM cells *in vitro* [11, 27]. In contrast, downregulation of PTEN accelerates the proliferation and migration of ASM cells [9, 28, 29]. In this study, our data demonstrated that PTEN expression was decreased in TNF-α-stimulated ASM cells. Functional assays revealed that restoration of PTEN expression markedly impeded TNF-α-induced proliferation and migration in ASM cells in vitro. In accordance with previous findings [8], our data confirmed that PTEN expression is decreased in the lung tissues of asthmatic, relative to healthy mice. Moreover, previous studies have reported that silencing of the PTEN gene accelerates airway remodeling in mice with allergic asthma [30], while PTEN upregulation suppresses airway remodeling in an OVA-induced murine asthma model [31]. In accordance with these findings, our study revealed that PTEN overexpression significantly prohibited airway remodeling in mice with asthma. Collectively, these findings confirm that PTEN acts as a negative regulator of airway remodeling in asthma. CD38 is ubiquitously expressed in ASM and its expression is enhanced in asthma [19]. CD38 deficiency remarkably impeded the airway hyperresponsiveness of asthmatic mice [20, 21]. Moreover, stimulation of TNF-α produced significant increases in CD38 expression in ASM cells, which enhanced the proliferation of ASM cells [32–34]. Our study consistently confirmed that CD38 was induced in ASM cells by TNF-α stimulation, and that CD38 knockdown inhibited, while CD38 overexpression promoted, the proliferation and migration of ASM cells induced by TNF-α stimulation. These findings suggest that CD38 may also play a key role in airway remodeling in asthma. As expected, we found that CD38 expression was upregulated in asthmatic mice and CD38 knockdown markedly decreased the airway remodeling that occurred in asthmatic mice. Therefore, our study suggests that CD38 is a crucial regulator of airway remodeling of asthma.

Interestingly, it has previously been reported that TNF-α-induced CD38 expression in ASM cells is regulated by PTEN [35]. Overexpression of PTEN has previously been shown to downregulate TNF-α-induced CD38 expression in ASM cells [35]. In this study, we demonstrated that PTEN expression decreased, while CD38 expression increased as a result of TNF-α-stimulation of ASM cells. Moreover, we showed that overexpression of PTEN markedly downregulated CD38 expression in TNF-α-stimulated ASM cells and in the OVA-induced mouse asthma model. Notably, overexpression of CD38] reversed PTEN-mediated suppressive effects on TNF-α-induced ASM cell proliferation and migration *in vitro* and on airway remodeling in asthmatic mice. Thus, our findings suggest that PTEN modulates airway remodeling in asthma through the regulation of CD38.

CD38 acts as a key regulator of intracellular Ca^2+^ levels in ASM cells [36]. In this study, we demonstrated that TNF-α stimulation elevated levels of intracellular Ca^2+^ in ASM cells. Functional experiments demonstrated that knockdown of CD38 markedly decreased, while CD38 overexpression increased, intracellular ASM cell Ca^2+^ concentrations. Increased concentrations of intracellular Ca^2+^ contributed to the upregulation of proliferation and migration in ASM cells and enhances airway remodeling in asthma [37, 38]. Moreover, we demonstrated that high levels of intracellular Ca^2+^ in the lung tissues of asthmatic mice were markedly decreased by knockdown of CD38. Collectively, our findings indicate that CD38-meidated Ca^2+^ signaling contributes to airway remodeling that occurs in asthma.

It has been reported that intracellular Ca^2+^ levels contribute to the activation of CREB [39–41]. Activation of CREB, in turn, enhances proliferation and migration in ASM cells [42, 43], which may indicate a role for CREB in airway remodeling. Herein we determined that TNF-α stimulation increased CREB phosphorylation in ASM cells. Moreover, phosphorylation of CREB was also upregulated in lung tissues of asthmatic mice. Notably, knockdown of CD38 impeded CREB phosphorylation in TNF-α-stimulated ASM cells *in vitro* and asthmatic mice *in vivo*. Therefore, our study indicates that CD38 contributes to the modulation of CREB activation in ASM cells. These findings are in accordance with previous studies [44] that demonstrated that CD38 promotes CREB activation via the modulation of intracellular Ca^2+^.

Certain limitation of our study should be noted. In this study, our data demonstrated that PTEN overexpression or CD38 silencing markedly attenuated the airway remodeling and airway inflammation of asthmatic mice *in vivo*. However, we did not directly assess where these observed changes in airway remodeling and inflammation were associated with changes in airway function. Therefore, further studies are needed to detect the regulatory effect of PTEN/CD38 axis on the airway function, such as airway hyperresponsiveness.

In conclusion, the findings of our study demonstrate that PTEN restricts airway remodeling that occurs in asthma through the downregulation of CD38-mediated Ca^2+^/CREB signaling. These data provided a detailed molecular mechanism for PTEN in the regulation of airway remodeling of asthma, and highlighted the key role of the PTEN/CD38/Ca^2+^/CREB signaling axis in the molecular pathogenesis of asthma. Therefore, details of this study provide novel molecular means, which can be exploited to develop improved therapeutic strategies for treating asthma.

## MATERIALS AND METHODS

### Experimental animals and ethics statement

Female, 8–10-week-old, BALB/c mice weighing 18–20 g were used in this study and were purchased from Hubei Provincial Laboratory Animal Center (Wuhan, China). Mice were housed in specific pathogen-free (SPF) facilities with free access to water and food throughout the experimental period. The use of animals for research purposes was approved by the Institutional Animal Care and Use Committees of Xi'an Jiaotong University (No. W2019001).

### Isolation, culture, identification and TNF-α stimulation of mouse ASM cells

ASM cells were isolated from the tracheal tissues of mice using the trypsin-collagenase combined digestion method. Mouse ASM cells were maintained using mouse ASM cell growth medium (Procell Life Science and Technology, Wuhan, China) containing smooth muscle cell growth supplements including 10% fetal bovine serum (FBS) and 1% penicillin/streptomycin mixed with 5% CO_2_ at 37°C. For the identification of ASM cells, cells were seeded into 12-well plates that contained slides. When cells reached 70–80% confluence, the slides containing cells were washed with phosphate buffer saline (PBS) and fixed with 4% paraformaldehyde for 15 min at room temperature and permeabilized using 0.5% Triton X-100 for 20 min at room temperature. Thereafter, cells were washed with PBS and incubated with goat serum (BOSTER Biological Technology, Wuhan, China) for 30 min at room temperature. Cells were incubated with an anti-α-smooth muscle actin (SMA) antibody (1:100; Abcam, Cambridge, UK) overnight at 4°C. Afterward, cells were incubated with a Cy3 Conjugated secondary antibody (1:100; BOSTER Biological Technology) for 1 h in the dark at room temperature. Cell nuclei were stained with DAPI (Beyotime, Shanghai, China) for 5 min in the dark at room temperature. Cells were observed and images were acquired using Olympus BX5 Biological Microscope (Olympus Corporation, Tokyo, Japan). For the induction of cell proliferation and migration *in vitro*, TNF-α (R&D Systems, Minneapolis, MN, USA) was added to ASM cells at a final concentration of 20 ng/ml and incubated for either 12 or 24 h [34, 45].

### Recombinant adenovirus construction, production and infection

The siRNA sequences targeting mouse CD38 (CD38-331: 5’-GAGCAUUUGUUUCCAAGAATT-3’ and 5’-UUCUUGGAAACAAAUGUUCTT-3’; CD38-509: 5’-GGGCUACAUUGCUGAUGAUTT-3’ and 5’-AUCAUCAGCAAUGUAGCCCTT-3’ and CD38-939: 5’-CCAUCGUGUAGACUUAAUATT-3’ and 5’-UAUUAAGUCUACACGAUGGTT-3’) and negative control (NC) siRNA (5’-UUCUCCGAACGUGUCACGUTT-3’ and 5’-ACGUGACACGUUCGGAGAATT-3’) were synthesized by GenePharma (Shanghai, China). The siRNA sequences of CD38-509 that most efficiently knocked down the gene were used to design CD38 shRNA using CD38-509-F: 5’-CCGGGGGCTACATTGCTGATGATTTCAAGAGAATCATCAGCAATGTAGCCCTTTTTTG-3’ and CD38- 509-R: 5’-GATCCAAAAAAGGGCTACATTGCTGATGATTCTCTTGAAATCATCAGCAATGTAGCCC-3’). The shRNA sequences were then subcloned into a pDC316-tdTomato-shRNA vector (Viraltherapy Technologies, Wuhan, China) containing *Age* I and *BamH* I restriction enzyme sites. The inserted sequences were confirmed to be correct via sequencing. The recombinant adenovirus expressing CD38 shRNA were produced using the AdMaxTM recombinant adenovirus packaging system. The coding sequences of CD38 cDNA (NM_007646.5) and PTEN (NM_008960.2) were synthesized and inserted into pDC316-mCMV-tdTomato vectors (Viraltherapy Technologies) with *Not* I and *Hind* III restriction enzymes sites. The inserted sequences were verified via sequencing. Recombinant adenovirus expressing either CD38 or PTEN were produced using the AdMaxTM recombinant adenovirus packaging system. ASM cells were infected with recombinant adenovirus expressing CD38 shRNA, the CD38 protein or the PTEN protein for 48 h and subsequently used for further experiments. Infection efficiency was determined by observing the fluorescence of red fluorescence protein (RFP) expressed from the adenovirus vector. The downregulation or upregulation of target genes was confirmed by Western blot.

### Real-time quantitative PCR (RT-qPCR) analysis

Total RNA was extracted using RIpure Reagent (Aidlab Biotechnologies, Beijing, China) and reverse transcribed to cDNA using HiScript Reverse Transcriptase (Vazyme Biotech, Nanjing, China) according to the manufacturers’ protocols. cDNA was detected using SYBR Green Master Mix (Vazyme Biotech) with a QuantStudio 6 Flex Real-Time PCR System (Applied Biosystems; Thermo Fisher Scientific, Waltham, MA, USA). The primer sequences used in this experiment were as follows: CD38 (242 bp) F: 5’-GTCCTGATCGCCTTGGTAGT-3’ and R: 5’-GGGGCGTAGTCTTCTCTTGT-3’; β-actin (240 bp) F: 5’-CACGATGGAGGGGCCGGACTCATC-3’ and R: 5’-TAAAGACCTCTATGCCAACACAGT -3’. PCR amplification was carried out following a cycling program that initiated at 95°C for 10 min and continued by repeating 40 cycles of 95°C for 30 s and 60°C for 30 s. Relative gene expression was calculated using the 2^−ΔΔCt^ method.

### Western blot analysis

Protein extraction from cultured cells or tissues was carried out using RIPA lysis buffer (Beyotime) containing 1 mM PMSF and a protease and phosphatase inhibitor cocktail (Beyotime). Protein concentrations were determined using a BCA Protein Assay Kit (Beyotime) in accordance with the manufacturer’s instructions. Equivalent amounts of proteins (40 μg) from each sample were separated via 15% sodium dodecyl sulfate polyacrylamide gel electrophoresis and then transferred to a Poly vinylidene fluoride membrane (Millipore, Billerica, MA, USA). The membrane was then immersed within a 5% nonfat-dried milk and Tris-Buffered Saline Tween-20 (TBST) solution for 2 h at room temperature. Afterward, the membrane was incubated with primary anti-CD38 antibodies (1:1000; Abcam), anti-PTEN antibodies (1:1000; Abcam), anti-CREB antibodies (1:500; Proteintech group, Wuhan, China), anti-phospho-CREB antibody (1:1000; Cell Signaling Technology, Danvers, MA, USA) or anti-β-actin antibodies (1:500; BOSTER Biological Technology). After an overnight incubation at 4°C, the membrane was washed with TBST and probed with horseradish peroxidase (HRP)-conjugated secondary antibodies (1:50000; BOSTER Biological Technology) for 2 h at 37°C. To develop protein bands, the membrane was incubated with enhanced chemiluminescence substrate (Applygen Technologies, Beijing, China) and exposed to x-ray film.

### EdU (5-ethynyl-2′-deoxyuridine) cell proliferation assay

The proliferation of ASM cells was assessed using an EdU cell proliferation assay using a BeyoClick EdU Cell Proliferation Kit with Alexa Fluor 488 (Beyotime). In brief, cells were incubated with a 10 μM EdU working solution for 2 h at 37°C. Cells were fixed using 4% paraformaldehyde and permeabilized with 0.5% Triton X-100. Cells were washed and then click additive solution at 0.5 ml × well^-1^. Cells were incubated at room temperature for 30 min in the dark. Positive cells were observed using an Olympus BX5 biological microscope (Olympus Corporation) using an excitation wavelength of 495 nm and emission wavelength of 519 nm.

### Cell migration assay

ASM cells were trypsinized and resuspended into serum-free medium and added to the transwell at 5×10^4^ cells × (200 μl × well)^-1^. The 24-well plate was filled with800 μl × well^-1^ 10% FBS medium. A transwell was added to the 24-well plate and cells were cultivated for 24 h at 37°C. Cells that had migrated to the transwell were fixed using 70% ice-cold ethanol for 1 h at room temperature. Cells were stained with 0.5% crystal violet dye for 20 min at room temperature. Cells were observed and images were acquired using a microscope.

### Calcium assay

Intracellular Ca^2+^ concentrations were measured using the Ca^2+^ fluorescent probe Fluo-3 AM (Beyotime). Fluo-3 AM is a type of fluorescent dye that can pass through cell membranes and bind to Ca^2+^ to produce strong fluorescence. In brief, 5,000 cells × well^-1^ ASM cells were seeded onto 96-well plates. After indicated treatment, 5 μM Fluo-3 AM indicator was added to ASM cells and cells were cultured for 30 min at 37°C in the dark. Cells were then washed with Ca^2+^-free PBS and incubated for 30 min at 37°C in the dark. Fluorescence intensity was measured using the FlexStation 3 multifunctional microplate reader (Molecular Devices, San Jose, CA, USA) with an excitation wavelength of 480 nm and emission wavelength of 530 nm.

### Establishment of an asthma model in mice

Mice were acclimatized in laboratory for seven days before model establishment. Twenty μg of ovalbumin (OVA; Sigma-Aldrich, Darmstadt, Germany) emulsified in 1 mg of aluminum hydroxide (Thermo Scientific, Waltham, MA, USA) in a total volume of 300 μl was used for subcutaneous injections into abdomen, left back and right back (100 μl per site) of mice on day 1, day 8 and day 15. On day 22, mice were challenged with an aerosol treatment of 5% ovalbumin (OVA) in saline using an ultrasonic nebulizer. Mice were challenged for 30 min per day for seven consecutive d. Recombinant adenovirus were administered intratracheally twice. Once on day 22 (one hour before OVA challenge) and once on day 23 (3 h after the previous OVA challenge). Seven days post-OVA challenge, mice were sacrificed, and their lungs were dissected for further analysis.

### Immunohistochemistry analysis

Lung tissues were fixed using 4% paraformaldehyde and were serially dehydrated, permeabilized and embedded in paraffin. The tissues were cut into 5-μm sections and mounted on slides. Slides were incubated at 60°C for 3 h, and twice dewaxed in xylene and serially rehydrated in ethanol. Slides were washed with PBS and stained with Mayer's hematoxylin and eosin (HE). To immunohistochemically detect α-SMA, CD38 and PTEN, slides were subjected to antigen retrieval in citrate buffer for 15 min, followed by cooling for 40 min at room temperature. Tissue sections were incubated using 3% hydrogen peroxide for 15 min at room temperature. Then, tissue sections were incubated with goat serum for 30 min at room temperature, and incubated with primary antibodies including the anti-α-SMA antibody (1:100; Abcam), anti-CD38 antibody (1:200; Abcam) and anti-PTEN-antibody (1:50; Abcam) overnight at 4°C. Sections were then incubated with the HRP-conjugated secondary antibody for 20 min at room temperature. Sections were washed with PBS and stained with diaminobenzidine (DAB) for color development. The sections were observed, and images were acquired using an Olympus BX5 biological microscope (Olympus Corporation).

### Statistical analysis

Quantitative results were expressed in the form of mean ± standard deviation (SD). Data were analyzed using GraphPad Prism 8 software (GraphPad Software, San Diego, CA, USA). The statistical differences were determined using either the Student’s *t* test or one-way analysis of variance (ANOVA) followed by a Bonferroni *post hoc* test. Differences were considered statistically significant when p < 0.05.
